# Mycotoxins Exposure of Lactating Women and Its Relationship with Dietary and Pre/Post-Harvest Practices in Rural Ethiopia

**DOI:** 10.3390/toxins15040285

**Published:** 2023-04-16

**Authors:** Addisalem Mesfin, Carl Lachat, Seifu Hagos Gebreyesus, Meselech Roro, Kokeb Tesfamariam, Tefera Belachew, Marthe De Boevre, Sarah De Saeger

**Affiliations:** 1 Centre of Excellence in Mycotoxicology and Public Health, Faculty of Pharmaceutical Sciences, Ghent University, 9000 Ghent, Belgium; 2 Department of Nutrition and Dietetics, Faculty of Public Health, Jimma University, Jimma 1000, Ethiopia; 3 Department of Human Nutrition, College of Agriculture, Hawassa University, Hawassa 1000, Ethiopia; 4 Department of Food Technology, Safety and Health, Faculty of Bioscience Engineering, Ghent University, 9000 Ghent, Belgium; 5 MYTOX-SOUTH^®^ International Thematic Network, Ghent University, 9000 Ghent, Belgium; 6 Department of Nutrition and Dietetics, School of Public Health, College of Health Sciences, Addis Ababa University, Addis Ababa 1000, Ethiopia; 7 Department of Reproductive Health and Health Service Management, School of Public Health, Addis Ababa University, Addis Ababa 1000, Ethiopia; 8 Department of Public Health, College of Medicine and Health Sciences, Ambo University, Ambo 1000, Ethiopia; 9 Department of Biotechnology and Food Technology, Faculty of Science, University of Johannesburg, Doornfontein Campus, Gauteng 2028, South Africa

**Keywords:** breast milk, multiple mycotoxins, fumonisins, pre/post-harvest

## Abstract

Mycotoxins can be transferred to breast milk during lactation. Hence, the presence of multiple mycotoxins (aflatoxins B1, B2, G1, G2, and M1, alpha and beta zearalanol, deoxynivalenol, fumonisins B1, B2, B3, and hydrolyzed B1, nivalenol, ochratoxin A, ochratoxin alpha, and zearalenone) in breast milk samples was assessed in our study. Furthermore, the relationship between total fumonisins and pre/post-harvest and the women’s dietary practices was examined. Liquid chromatography coupled with tandem mass spectrometry was used to analyze the 16 mycotoxins. An adjusted censored regression model was fitted to identify predictors of mycotoxins, i.e., total fumonisins. We detected only fumonisin B2 (15% of the samples) and fumonisin B3 (9% of the samples) while fumonisin B1 and nivalenol were detected only in a single breast milk sample. No association between total fumonisins and pre/post-harvest and dietary practices was found (*p* < 0.05). The overall exposure to mycotoxins was low in the studied women, although fumonisins contamination was not negligible. Moreover, the recorded total fumonisins was not associated with any of the pre/post-harvest and dietary practices. Therefore, to better identify predictors of fumonisin contamination in breast milk, longitudinal studies with food samples in addition to breast milk samples and with larger sample sizes are needed for the future.

## 1. Introduction

Mycotoxins are toxic secondary metabolites produced by specific fungal genera, mainly *Aspergillus*, *Penicillium*, *Alternaria*, and *Fusarium*. Aflatoxins (AFs), fumonisins (FUMs), trichothecenes, zearalenone, ochratoxin A, and patulin are the most concerning. Mycotoxins significantly affect both human and animal health, and the economy of the agriculture–food system [1]. 

Aflatoxin B1 undergoes hydroxylation to form aflatoxin M1 (AFM1) metabolites, which are excreted in the milk of lactating mammals due to the consumption of Aflatoxin B1 contaminated food or feed [2]. AFM1 has been classified by the International Agency for Research on Cancer (IARC) as “carcinogenic to humans” [3]. Though poorly understood, other mycotoxins can also potentially be excreted from the dietary intake of mothers to breast milk during lactation [2]. AFM1 and ochratoxin A are the most studied mycotoxins in breast milk [4]. Aflatoxin B1 and its metabolite AFM1 seem to be the main ones found in breast milk in Sub-Saharan Africa [5]. The *Aspergillus* fungi which produce AFs and ochratoxin A mainly occur after harvest, and they are also called storage-related mycotoxins [6]. Although *Fusarium* mycotoxins are reported to be highly abundant in food crops in Africa (deoxynivalenol (81%), FUMs (51%) and zearalenone (44%) [7], they are less studied in breast milk [8]. A study in Tanzania reported fumonisin B1 (44.3%) in breast milk samples [9]. *Fusarium* mycotoxins are generally categorized as field-derived mycotoxins since *Fusarium* fungi usually arise at the pre-harvest time of agricultural production [6].

A comprehensive and easy actionable guide of pre-harvest and post-harvest factors that focus on the prevention and control of mycotoxins in grains has been framed by experts in five key areas: (1) sustain plants’ vigor and health; (2) reduce toxigenic fungal populations in growing plants and storage; (3) rapidly reduce the moisture content of grains and avoid rehydration; (4) safeguard husks/hulls or pericarp/testa; and (5) clean and remove mycotoxin high-risk components [10]. Food processing can further reduce mycotoxin levels through physical removal and decontamination [11]. Moreover, diversifying diet lowers the risk of mycotoxin exposure by decreasing the intake of common mycotoxin-contaminated staple foods such as maize [12]. 

The dependence of African diets on monotonous cereals increases mycotoxin exposure [13]. The risk is even higher in Sub-Saharan Africa due to frequent and multiple contaminations of mycotoxins in staple diets [5]. Similarly, crops such as teff, wheat, maize, sorghum, barley, and groundnut in Ethiopia have also been found to be contaminated with *Aspergillus* and *Fusarium* mycotoxins [14]. Although national data on the human health implications of mycotoxins exposure are missing in Ethiopia, the prevalence data generated so far indicate a possible public health risk [14,15]. 

In Ethiopia, knowledge of fungi and mycotoxins is generally minimal. Poor household storage and processing practices of staple foods and intentional adulteration by traders are common [14,16]. Moreover, little effort has been exerted in the control of mycotoxins except for the regulation of very few commodities by the Ethiopian Standard Authority [17]. 

Applying validated biomarkers for mycotoxins’ internal exposure assessment in humans is a reliable approach as it encloses all sources of contamination and decreases uncertainties [18,19]. To date, studies conducted in Ethiopia have focused on food crops and mainly on AFs while not covering other mycotoxins such as FUMs in a multi-mycotoxin approach. Hence, studies on bio-monitoring of mycotoxins such as in breast milk are lacking except a study reported by Mesfin et al. (2020) on AFs exposure among lactating women in the Sidama region [20]. Moreover, though the importance of good agricultural practices has been indicated in previous studies from Ethiopia [14,17,20,21], the effects of maize agronomic practices, storage conditions, and dietary practices on mycotoxins exposure among lactating women have not been reported.

The present study examined the exposure to multiple mycotoxins in the breast milk samples of lactating women in the Meskan and Mareko districts in southern Ethiopia. Moreover, the study examined the relationship between total FUMs and maize pre/post-harvest and the women’s dietary practices.

## 2. Results

### 2.1. Socio-Demographic Characteristics of the Lactating Women 

A total of 138 lactating women participated in the study, of whom 99.3% were married, with a mean age of 27.4 ± 5.2 years. Just under half (48.6%) of the participants had a formal education, whereas 21.7% were able to read and write and 29.7% had no formal education. The median family size was 5 ± 4.6, and agriculture was the major source of income (68.1%), followed by small personal business (18.1%). The majority (72.4%) of the household harvested crops were reported to be maize (93.4%) followed by teff (37.3%) and wheat (13.1%).

### 2.2. Mycotoxin Exposure in the Lactating Women 

Except for FUMs and nivalenol, the other mycotoxins, i.e., aflatoxins B1 (LOD = 0.20, LOQ = 0.39), B2 (LOD = 0.37, LOQ = 0.75), G1 (LOD = 0.31, LOQ = 0.62), G2 (LOD = 0.23, LOQ = 0.46), and M1 (LOD = 0.38, LOQ = 0.77); alpha (LOD = 3.00, LOQ = 6.00) and beta (LOD = 2.70, LOQ = 5.40) zearalanol; deoxynivalenol (LOD = 3.01, LOQ = 6.02), hydrolyzed fumonisin B1 (LOD = 2.42, LOQ = 4.85), ochratoxin A (LOD = 0.66, LOQ = 1.32), ochratoxin alfa (LOD = 0.47, LOQ = 0.95), and zearalenone (LOD = 2.19, LOQ = 4.38), were not detected (<LOD). The FUMs contaminated 16% of the total samples; of which the most prevalent contamination was due to FB2 (15%), followed by FB3 (9%). The contamination by FB1 and nivalenol was negligible with only 1% of the samples (Table 1).

### 2.3. Dietary Practices and Food Security Status 

Maize was found to be the most frequently consumed crop by all (100%) of the women. The majority (79.7%) consumed maize more than once per day, and the rest consumed it once per day (5.8%) or 3–6 times per week (10.9%). Just over half (52.2%) of the study women reported that they never consumed sorghum. Of the other half who consumed sorghum, only a few of them reported that they consumed it more than once per day (7.2%) and once per day (1.4%). Instead, 21.7% reported that they consumed sorghum twice or less per month, and 10.9% reported this figure as being 3–6 times per week. The majority (84.8%) of the study women reported that they never consumed millet. A minority of the women (8.7%) consumed millet twice or less per month. None of the women reported that they consumed millet on a daily basis. 

Regarding the food security status of the households, only 23.1% of the households of the study women were food secure but the rest (76.8%) were experiencing different levels of food insecurity: mild (20.2%), moderate (37.7%) and severe (18.8%).

### 2.4. Food Groups Consumed by the Lactating Women

The food groups consumed by the lactating women are shown in Table 2. Higher consumption of grains, white roots, and tubers was observed (98%) in contrast to approximately non-consumption (1%) of nuts and seeds. Higher consumption of non-green vegetables (80%) and green vegetables (70%) was also reported compared to a relatively low consumption of eggs (8%) and meat, poultry, and fish (7%). Nearly half (46%) of the women consumed dairy products. Accordingly, most (77%) of the study women demonstrated a lower dietary diversity score (<5 food groups).

### 2.5. Knowledge of the Lactating Women about Mycotoxins

Most (66.7%) of the women had the knowledge that high temperatures lead to mold formation, whereas just over half (52.9%) mentioned moisture as a cause and some (11.5%) of them also mentioned the use of expired insecticides as a possible cause. 

Moreover, the knowledge of the women about the health effects of consuming mycotoxin contaminated foods/crops was investigated whereby approximately half reported that they did not know the health effects. The other half of the women gave varied responses that mycotoxins induce diarrhea (35.8%), vomiting (16.4%), and growth impairment (8.2%).

Buying moldy grains of maize or other crops was not common as the majority (95%) of the women demonstrated good knowledge that they do not buy such crops from the market. Additionally, the respondents were brought to their attention which crops they think are more susceptible to mold spoilage. The majority (90.6%) of the women reported cereals as the most susceptible crops to mold spoilage compared to other crops. Furthermore, they recalled the types of cereals that they think are more prone to mold formation, and majorly, they mentioned maize (90.5%), followed by sorghum (37.7%) and wheat (29%).

### 2.6. Pre/Post-Harvest Practices Concerning Maize 

The maize-related pre/post-harvest practices of the study households are presented in Table 3. From the survey, no other watering method for maize fields other than rainwater (100%) was mentioned. Weeding the maize field (97%), cleaning residues from the previous harvest (91%), and harvesting maize when it becomes completely dry (89%) were also reported by the majority of the women. Moreover, more than half (56%) of the women revealed that they rotate crops to avoid growing the same crop. The application of insecticides/fungicides in the maize growing field was the least (23%) reported pre-harvest practice.

For the post-harvest practices, sorting out undesirable maize grains before storage was largely (98%) practiced, followed by decontamination of moldy maize grains (81.1%), disinfecting the storage area before storing maize (71%), and the use of pesticides in storage (66.7%). However, fumigation as one of the storage cleaning methods was less practiced (10.8%). 

Predominantly (60.1%), a woven polypropylene bag locally called “madaberia” was mentioned as maize storage material used, and only 16.6% reported that they use local storage made from wood or bamboo. Most importantly, the majority (86%) of the families of the study women store maize from four months up to one year. 

### 2.7. Traditional Household Maize Processing Practices in Flour Preparation 

Sorting out visibly moldy maize grains in preparation for maize flour was a common practice reported by almost all (97.8%) of the women. In contrast, only 10.9% practice dehulling, similarly only 10.9% practice soaking/washing, and almost none (0.7%) of the women reported to practice roasting maize grains in flour preparation. Even more, the women were asked what they do with the undesirable grains removed after the sorting and dehulling of maize grains. Unfortunately, most (71.7%) of them reported that they use it for feed, others (31.1%) reported it for making beverages, and only a few (3.6%) of them declared that they burn it. 

### 2.8. Predictors of Total FUMs Exposure in Breast Milk Samples of the Lactating Women

Only FUMs were considered for further statistical analysis since the rest of the mycotoxins were not found (<LODs), except a single sample was contaminated with nivalenol. Moreover, as only one sample was contaminated by FB1, the total FUMs for the remaining 137 samples was computed as a summation of FB2 and FB3 only.

The results of the univariate regression between each PC and total FUMs are depicted in Table 4. Three PCs were retained from the PCA of the pre-harvest practices which represented 54% of the total variance. Frequency of weeding and use of insecticide in the field were the variables embodied in PC1; variety common name were used in PC2 and removing residues from previous harvest were used in PC3. Crop rotation, sensory methods used for harvesting maize, and special drying method applied were the variables not represented in the first three selected PCs of the pre-harvest group.

Similarly, the PCA for post-harvest practices was represented by the first three PCs explaining 59% of the total variance. The variable storage condition was denoted in PC1, the use of pesticides in storage contributed to PC2, and decontamination of molds was represented in PC3. The variables, i.e., disinfecting storage, use of fumigation in storage, type of storage material, and duration of storage were not retained in the first three PCs. 

The PCA of mycotoxin knowledge was represented in five PCs. The total variance explained by the PCs was 67%. PC1 contained two variables i.e., crops prone to mold spoilage and maize prone to mold spoilage. Mycotoxin causes diarrhea was the variable that contributed to PC2. Other health effects of mycotoxin were encompassed in PC3. Wheat prone to mold spoilage is represented in PC4 and moisture causes mold in storage is represented in PC5. Do not know the health effect of mycotoxins, use of expired insecticides causes mold in storage, high temperature causes mold in storage, other causes of mold in storage, have heard about fungus affected maize, mycotoxin causes vomit, mycotoxin causes growth impairment, buy moldy crops, and sorghum prone to mold spoilage were the variables not represented in the five PCs.

Two PCs were retained from the PCA of the maize household processing method which explained 57% of the total variance. Make beverages from maize removals and make other things from maize removals were the variables represented in PC1 and PC2, respectively. Variables maize removals use as feed, dehull and soak maize before making flour were not retained. 

The univariate analysis showed that none of the PCs were associated with total FUMs (*p* ≥ 0.05). However, after adjusting for confounders in the multivariate model, the age of the women showed a statistically significant association with total FUMs (*p* < 0.034). A one-year increase in age showed on average a 1.21 ng/mL decrease in FUMs level.

## 3. Discussion

Our study assessed multiple mycotoxins in breast milk (i.e., 16 biomarkers) among lactating mothers and examined the relationship between total FUMs and maize pre/post-harvest and the women’s dietary practices. Our finding indicated that FUMs contaminated 16% of the total samples. The FUMs’ contamination levels ranged from <LOD to 32.5 ng/mL. 

Although the synergy or antagonism interaction of mycotoxin mixtures is still not fully understood, synergy among *Fusarium* toxins has been highlighted in studies [22,23]. AFM1 is the most studied mycotoxin in breast milk, while there are limited studies on FUMs and other mycotoxins. Therefore, we were unable to find studies reporting FUMs’ contamination in breast milk samples from Ethiopia. However, *Fusarium* mycotoxins such as FUMs were documented in Ethiopian agricultural products [14] and in blood samples from two studies. Compared to our finding, one of the studies in serum was conducted in the same study area of ours’ by another Butajira Nutrition, Mental Health, and Pregnancy (BUNMAP) Cohort study research team and reported a higher prevalence of FUMs exposure (FB2 (98.8%), FB3 (95.3%), and FB1 (93.3%)) [24]. The other study in serum samples of 6–35 months children in Ethiopia reported a comparable prevalence of FB3 (11%) to our finding but in contrast to the prevalence of FB2 (0%) and FB1 (2%) [25]. 

Additionally, studies from Tanzania and Brazil analyzed FB1 contamination in samples of breast milk. The Tanzanian investigation found 44.3% of FB1 at concentrations ranging between 6.57 to 471.05 ng/mL (LOD = 5.5 ng/mL) [9]. The relatively higher LOD reported in that study may further indicate that the concentrations in the positive samples were higher. On the other hand, the study from Brazil found FB1 (LOD = 2.1 ng/mL) contamination only in two samples (3%) with concentrations of 2.2 and 3.4 ng/mL [26], which is comparable to our finding.

The most likely explanation for the occurrence of FUMs in our study might be related to the women’s reported high consumption of maize (100%) and low dietary diversity (77%). It has been determined that maize crops are more susceptible to FUMs contamination [27]. Moreover, a less diversified diet increases the risk of mycotoxin exposure, particularly in low-income settings [28,29]. 

As late harvesting favors the occurrence of FB1 and FB2 [30], the other reason for the observed FUMs in our study might also be related to the majority (89%) of the study households allowing maize crops to overly dry on the field before harvesting. 

However, the level of FUMs’ contamination recorded in our study was not immense. The abundant good pre/post-harvest practices reported by the women might explain the low magnitude of FUMs observed in our study compared to the other studies reported elsewhere. Moreover, the variation in breast milk sampling might also be another reason for the observed difference [9,26]. Numerous studies have indicated that good pre/post-harvest practices play a significant role in reducing mycotoxins contamination [31,32].

To date, evidence indicates that FUMs are not acutely toxic but there are several studies covering their chronic toxicity and carcinogenicity in animals; yet, their health impact on humans is unclear [33,34]. Biomarker-based epidemiological studies of FUMs [33] with robust experimental research and adequate sample size are still required [34]. Even though the threat of FUMs is not explicitly evidenced in humans, studies have shown that their exposure increases the risk of cancer, neural tube defects, and other birth defects [3,35]. Therefore, the consumption of FUMs by infants through contaminated human breast milk should not be ignored [35]. 

Unexpectedly, none of the breast milk samples in our study were contaminated with Afs, hydrolyzed fumonisin B1, deoxynivalenol, zearalenone, ochratoxin A, ochratoxin alpha, alpha, and beta zearalanol. Regarding AFM1, our result is consistent with studies conducted in Angola (LOD = 0.005 ng/mL) [36] and Brazil (LOD = 0.02 ng/mL) [26] in which AFM1 was not detected in breast milk samples. In Ethiopia, we found only one study which assessed AFM1 in 360 breast milk samples collected in two seasons. AFM1 was detected in 64.4% (LOD = 0.005 ng/mL) of the samples that ranged from undetectable to 0.143 ng/mL [20]. Though no other published studies on mycotoxins in breast milk from Ethiopia exist, another survey in the Sidama zone, Ethiopia assessed urinary AFM1 levels in lactating women. The study revealed that AFM1 (LOD = 0.00015 ng/mL) exists in 53.3% of the samples (undetectable to 2.6 ng/mL) [37]. Moreover, from the same study area as ours, 62.4% AFM1 at concentrations ranging from 0.15 to 0.4 ng/mL (LOD = 0.00015 ng/mL) in urine samples among children 12–59 months of age was reported [38]. Moreover, urinary AFM1 among school-age children (93%) [39], pregnant women (72.5%) [40], and young children (7%) [41] has also been reported in Ethiopia. Furthermore, studies conducted in Lebanon (93.8%, range: 0.0002–0.0079 ng/mL, LOQ = 0.0002 ng/mL) [42], India (41%, range: 0.0039–1.2 ng/mL, LOD = 0.0078 ng/mL) [43], Ecuador (13%, range: 0.053–0.458 ng/mL, LOD = 0.033 ng/mL) [2], and Guatemala (5%, range: 0.004–0.333 ng/mL, no LOD) [44] also consistently reported AFM1 in breast milk samples. This discrepancy might be due to the fact that the above-mentioned studies reported a much lower LOD and LOQ compared to our study. This may also imply that the concentrations reported in the studies might not be high.

In addition, contrary to our findings, other mycotoxins such as beauvericin, alternariol monomethyl ether, ennaintin, ochratoxin A and B, sterigmatocystin, zearalenone, aflatoxin B2, G2, and M2 have been intermittently reported in breast milk by several studies [26,43,45,46,47].

Differences in mycotoxin concentrations between our study and others may be attributable to differences in analytical equipment performance including the LOD and LOQ, biological matrix analyzed, breast milk sampling method, sample size, and pre/post-harvest methods. 

The different analytical approaches used in various studies might explain the differences in the mycotoxins detected. The specificity of analytical equipment used in detecting mycotoxins in human milk has been questioned [8]. LC-MS/MS offers great promise in terms of specificity and accuracy of analyte identification, but ELISA methods are less specific [8,48]. 

Some of the studies compared with our findings monitored mycotoxins in urine samples and others in serum samples. Urinary mycotoxin exposure shows recent exposure depending on the type of food consumed. Instead, breast milk is a complex matrix and transfer rates from diet appear to be lower. For instance, the FB1 consumed may be retained and excreted in biological fluids before getting to breast milk [9]. Determining mycotoxin levels both in maternal blood plasma and in milk is the prime approach to estimate lactational transfer in humans [49]. Except for aflatoxins and OTA, data on the lactational transfer of most mycotoxins including FUMs are insufficient [8,45,49,50]. 

Moreover, the difference between our findings and others could be due to variations among the studies in breast milk sampling. Some of the studies focused on early lactation, while others targeted lactating mothers of 6 months and onwards. Other factors such as the timing of the day, hind or fore milk, and the order that an infant is born in a family may introduce a difference in the stability of toxins and may affect the mycotoxin excretion in breast milk [8,49,51]. 

On top of the sampling, the variation in the sample sizes accounted for and their respective power between our study and others might affect the proportion of the mycotoxin detection. Though we included all lactating women found in the study areas during our survey time, the limited number of lactating women found may underpower our study. 

On the other hand, the good pre/post-harvest practices reported by the women might have played a significant role in controlling mycotoxin production or decontaminating the food crops [31]. This might imply that the chance for fungi development and production of their respective mycotoxins in the staple food, i.e., maize, consumed by the women has been minimized. On top of this, the reported practice of sorting moldy maize grains (97.8%) might have also meaningfully reduced the chance of consuming mycotoxin-contaminated maize crops by the women [28]. Our report shows that the sorted-out maize grains were mainly used for feed (71.7%). However, the chance of the women consuming mycotoxins transferred from contaminated feed [52] might have decreased due to the low consumption of milk and milk products (46%) and other animal products, i.e., meat (7%) and eggs (8%). On top of that, weather variability and the subsequent agricultural practices observed between and within seasons in Ethiopia might make mycotoxin prediction difficult [53]. 

The regression analysis provided no evidence for a statistically significant association between total FUMs and women’s pre-and post-harvest and dietary practices. Instead, the age of the women was found to be a predisposing factor to FUMs’ occurrence in the breast milk samples. The few studies on FUMs occurrence in breast milk provided no information on the contributing factors. Thus, a comparison between our findings with these studies cannot be sought. We suggest further similar studies to perform multiple regression analysis considering more confounding variables.

Lastly, despite the absence of occurrence of the common mycotoxins in our study, the knowledge gap observed among the lactating women on some of the basic understandings about mycotoxins was not negligible. For instance, approximately half of the mothers did not consider the health implications of mycotoxin consumption. Thus, disseminating information to communities on ways how to manage moldy foods and feeds and preventing mycotoxin exposure is important [16].

### Strengths and Weaknesses of the Study

We analyzed 16 mycotoxins in breast milk samples. Moreover, we used LC-MS/MS which is a highly specific analytical technique in detecting mycotoxins at low concentrations. However, as a multi-mycotoxin method was used, LODs varied from one mycotoxin to another and reduced the sensitivity. Data from longitudinal studies are suitable to capture temporal sequences and generate cause and effect relations [54]. Thus, the cross-sectional survey we employed was unable to examine the stage of lactation and seasons as possible factors for mycotoxins occurrence in the breast milk samples. It is likely that the relatively small sample size used in our study might have also influenced the statistical power and the subsequent statistical inferences, i.e., the coefficients and *p*-values of the regression result [55,56,57]. Moreover, we sampled only breast milk and tried to associate the mycotoxin result with dietary and pre/post-harvest practices recall data. Analysis of mycotoxins and fungi in food sample data which is the key factor between the biological sample and pre/post-harvest practices is lacking. Such data could have partially explained the FUMs finding. Due to these limitations, the findings of our study should be interpreted with caution. 

## 4. Conclusions

No significant exposure to most of the mycotoxins was recorded, in exception to modest exposure to FB2 and FB3. Irrespective of the absence of common mycotoxins, however, limited knowledge among the study participants about mycotoxins are warranted. This stresses the need for mycotoxin sensitization of the households in the study area. We ruled out a possible association between the ages of the women with FUMs exposure; however, this was not the case for any of the dietary and pre/post-harvest practices. Hence, it is evident that further studies including food sample data besides the breast milk samples with larger sample sizes and across different stages of lactation are required. 

## 5. Materials and Methods

### 5.1. Study Design and Location 

We conducted a community-based cross-sectional survey nested in the BUNMAP. BUNMAP is a mother–child cohort study of Addis Ababa University, Ethiopia. The study site was the Butajira Health and Demographic Surveillance Site of Meskan and Mareko districts, Gurage zone, Southern Nations and Nationalities and Peoples Regional State, Ethiopia. The Butajira Health and Demographic Surveillance Site is one of the oldest surveillance sites in Africa established in 1986 which consists of nine rural villages and one urban village of different ecological zones. A cohort study within the BUNMAP examined multi-mycotoxins in serum samples of mothers during their pregnancy period. The study reported that the pregnant mothers were co-exposed to at least five mycotoxins, of which FUMs and tenuazonic acid were the most frequently detected [24]. Our current study extended and assessed multiple mycotoxins among the same mothers during their lactation period. 

### 5.2. Study Subjects and Sampling

The study included all lactating women who had infants between three and five months during the study period. We recruited 138 mother–infant dyads using the BUNMAP open cohort. 

### 5.3. Data and Sample Collection

#### 5.3.1. Measurements 

The data were collected and supervized by the Butajira Health and Demographic Surveillance Site experienced data collectors and supervisors. Training was given for data collectors and the questionnaire was also pre-tested before use. The understandability of some questions was rectified as per the feedback received from the pretest. The questionnaire-based data were electronically collected using Open Data Kit (ODK). Completeness of the data was checked by the supervisors at the end of each interview and final checkup was ensured by the principal investigator before submission to the central database. 

The five sections of the questionnaire were: socio-demographic and economic information; dietary diversity and food frequency of the lactating women; household food insecurity; pre/post-harvest practices; and household food processing methods with regard to maize. The socio-demographic and economic questionnaire was adapted from the previous questionnaire developed by the BUNMAP study group during the baseline survey. The Minimum Dietary Diversity for Women developed by the Food and Agricultural Organization was used to assess the food groups consumed by the lactating women 24 h before the survey time [58]. The number of food groups consumed by the women out of 10 food groups was recorded. The frequency of consumption of mycotoxin-prone foods, i.e., maize, sorghum, and millet, were qualitatively assessed using adapted food frequency questionnaires (FFQ) over a reference period of one month [59]. 

The Household Food Insecurity Access Scale (HFIAS) was used to assess the food security status of the respondents’ households [60]. The questionnaire contains nine occurrence questions, and each of the nine questions were followed by frequency-of-occurrence questions with a recall period of one month. A higher cumulative score of HFIAS indicates an increased possibility of food insecurity. The HFIAS guideline summarize the cumulative HFIAS score into four levels of household food insecurity: food secured, mild, moderate, and severe food insecurity.

Questions on pre/post-harvest practices related to maize, knowledge about mycotoxins, and household maize processing techniques were adapted from questionnaires used by previous similar studies [21,61]. The women were interviewed about the maize pre/post-harvest practices employed by their family as women in Ethiopia engage in the majority of the agricultural activities.

#### 5.3.2. Breast Milk Sample Collection 

The women themselves hand expressed ≥25 mL of breast milk into sterile 50 mL falcon tubes. Each woman was instructed on how to manually hand express and provide a sample depending on the availability of breast milk. The samples were immediately kept in ice boxes, and, at the end of each data collection day, they were transferred to −20 °C at the local health center. The samples were transported from the study site to the Ethiopian Public Health Institute at Addis Ababa, Ethiopia, and kept at −40 °C until they were shipped to Belgium. The samples were then shipped in dry ice to the Centre of Excellence in Mycotoxicology and Public Health, Faculty of Pharmaceutical Sciences, Ghent University, Belgium, and stored at −80 °C until analysis. All breast milk samples were thawed prior to extraction for the determination of mycotoxins. 

### 5.4. LC-MS/MS Analysis of the Breast Milk Samples

The breast milk samples were quantitatively analyzed for 16 different mycotoxins. We used an extraction method and the LC-MS/MS method with the XEVO TQ-S (Waters^®^, Manchester, UK) that has been validated and published previously by a research team in our laboratory (LOD (0.20–3.40 ng/mL), relative standard deviation intra-day (2.25–14.09%) and inter-day (1.60–17.46%) precisions, apparent recovery (86.67–118.43%), and linearity (≥0.991 R^2^) [62]. 

One mL of milk was added to a fifty mL centrifuge tube. Internal standards, i.e., 40 µL of a 1 µg/mL deepoxy-deoxynivalenol (DOM) solution (50 µg/mL stock diluted 50 times in MeOH) and 40 µL of a 1 µg/mL zearalanone (ZAN) solution (1 mg/mL stock diluted 1000 times in ACN) were added. For the control spike, 50 µL of multi-mycotoxin standard mixture was added. The samples were then incubated in the dark for 15 min. Subsequently, 4 mL ACN/formic acid (99/1 *v*/*v*) was added and vortexed for 30 s. Next, the samples were shaken on the overhead shaker for 10 min, followed by centrifugation at 3000 mg for 10 min. Afterwards, an OASIS^®^ PRiME HLB column (6 cc (200 mg) extraction cartridges, part no WAT106202, WatersTM, Ireland) was conditioned with 3 mL ACN/formic acid (99/1, *v*/*v*). The supernatant was then transferred to the Oasis PRiME HLB cartridge (60 mg/3 cc) and the eluate was collected in a glass test tube. The samples were evaporated at 40 °C under a gentle nitrogen flow. An injection solvent was prepared by adding 50 mL MeOH LC-MS grade to a 100 mL volumetric flask, and then the volumetric flask was filled with water up to the mark and homogenized. The dry residue was then dissolved in 200 μL of the water/MeOH (50/50 *v*/*v*) injection solvent and vortexed. Afterwards, 200 µL n-hexane was added, vortexted, and then centrifuged at 3000× *g* for 2 min. The samples were then passed through a Millipore filter (0.22 μm) and ultra-centrifuged (10,000 rpm for 10 min). Subsequently, 100 µL of the lower phase was transferred into a vial and any air bubbles were removed. Finally, the samples were analyzed using the Waters^®^ Acquity UPLC (column HSS T3 1.8 μm) system coupled to a Quattro XEVO TQ-S mass spectrometer. 

Mobile phase A: water/methanol/acetic acid (94/5/1, *v*/*v*/*v*) + 5 mM ammonium acetate, and mobile phase B: water/methanol/acetic acid (2/97/1, *v*/*v*/*v*) + 5 mM ammonium acetate were used. The gradient elution program started at 95% mobile phase A, and mobile phase B increased gradually to 65% in 7 min. Then, mobile phase B increased gradually to 99% in 13 min. The total run time was 18 min. The mass spectrometer was operated in the positive (ES+) electrospray ionization mode. The presence of mycotoxins in a sample was confirmed according to the European Commission Decision (EC) No. 2002/657 recommendations: having 3 or more identification points; a signal to noise ratio of >3; the use of relative ion intensity and relative retention time [63]. MassLynx version 4.1 and TargetLynx version 4.1 (Waters^®^, Manchester, UK) software was used for data acquisition and processing. The limits of detection (LOD) and the limits of quantification (LOQ) are reported in Table 1. The samples were considered positive with any of the mycotoxins if the concentrations were above the LOD.

### 5.5. Data Management and Analysis

Questionnaire-based data collected using ODK was transferred from a server to be analyzed in R version 4.0.5 (31 March 2021) [64]. The data were cleaned by identifying missing values and outliers. A Shapiro–Wilk test of normality was carried out to check if the data were normally distributed using a *p*-value > 0.05 implying that the distribution is normal. Descriptive analysis of frequency and percentage for categorical variables was used. The mean and standard deviation for the normally distributed data and the median and interquartile ranges (IQRs) for the non-normally distributed data were used to describe the continuous variables.

Tabulation of Minimum Dietary Diversity for Women was carried out by adding one point if any food in the 10 food groups was consumed and summed into a score ranging from 0 to 10. Each woman was then coded yes or no for scoring at least ≥5, followed by calculation of the proportion of women who score at least ≥5 [58].

The HFIAS was scored first by coding the frequency-of-occurrence as 0 for all cases where the answer to the corresponding occurrence question was “no” and then summing the codes for each frequency-of-occurrence question. The household response to the nine frequency-of-occurrence questions was coded into three levels as rarely, sometimes, and often. The maximum score for a household is 27. Finally, the households were categorized into food secure, mildly food insecure, moderately food insecure, and severely food insecure as described in Coates et al. (2007) [60].

We computed total FUMs by summing up FUM B1, B2, and B3 as these are the major FUMs grouped together. The Shapiro–Wilk test showed that the total FUMs data were not normally distributed (*p*-value < 0.05) due to left censoring of 85% of the data. A censored regression model was hence used [65]. 

Four major categories of variables (pre-harvest practices, post-harvest practices, mycotoxin knowledge, and maize household processing methods) were considered as predicting variables. To avoid over adjustment and multi-collinearity across the pre/post-harvest practices, a data reduction approach was applied to summarize these agricultural practices. For this purpose, we applied principal component analysis (PCA) with varimax rotation to generate latent factors explaining the variance within each pre/post-harvest practice [66]. The amount of variance carried in eigenvalues and scree plots was used to decide the number of principal components (PC) to be retained. A PC with an eigenvalue < 1 was not retained. After selecting the PCs, the contribution of the original variables in a PC was inspected using values of varimax rotated loadings. Loadings < 0.1 were blanked and any variable that showed a larger loading was regarded as a contributor to that PC. Those variables which did not appear in the retained PCs were not represented in the subsequent regression analysis. Finally, scores from the newly constructed PCs were generated for the 138 samples and used for the regression analysis. Univariate regression model between each PC and the total FUMs was first fitted, and further multivariate regression analysis was executed including all of the PCs and confounders, i.e., FFQ maize, dietary diversity, the age and educational level of the women, and households’ food security status. A *p* value of 0.05 was taken as the significance level. The fitness of the models in terms of log-likelihood was compared and the multivariate model showed better model fitness (log-likelihood −117) than the univariate models (log-likelihood −124 to −126). 

## Figures and Tables

**Table 1:** Mycotoxins in the breast milk samples of the lactating women (*n* = 138).

Mycotoxin ^1^	LOD ^2^ (ng/mL)	LOQ ^3^ (ng/mL)	N (%) of Positive Samples	Maximum (ng/mL)
NIV	2.78	5.57	1 (1)	2.81
FB1	3.40	6.80	1 (1)	8.26
FB2	1.99	3.98	21 (15)	24.98
FB3	2.38	4.76	12 (9)	7.52
Total FUMs	-	-	22 (16)	32.51

^1^ FB1 (fumonisin B1), FB2 (fumonisin B2), FB3 (fumonisin B3), NIV (nivalenol), and total FUMs (total fumonisins). ^2^ LOD limit of detection; the samples were considered positive if values were equal to or above the LOD. ^3^ LOQ limit of quantification.

**Table 2:** Food groups consumed by the lactating women (*n* = 138).

Food Groups ^1^	*n* (%)
Grains, white roots and tubers, and plantains	135 (98)
Pulses	40 (29)
Nuts and seeds	1 (1)
Milk and milk products	63 (46)
Meat, poultry, and fish	10 (7)
Eggs	11 (8)
Dark green leafy vegetables	97 (70)
Vitamin A-rich fruits andvegetables	26 (19)
Other vegetables	110 (80)
Other fruits	21 (15)

^1^ The FAO food groups of minimum dietary diversity for women was used.

**Table 3:** Pre/post-harvest practices of maize among households of lactating women (*n* = 138).

Variables	Categories	*n* (%)
*n* = 100		
Residues cleared from field	Yes	91 (91)
	No	9 (9)
Maize variety used	“Shone-pioneer”	35 (35.3)
	“extension”	26 (25.8)
	“mirit zer” ^1^	10 (10)
	“BH660”	14 (13.9)
	Others	15 (15)
Field watering method	Rainwater	100 (100)
Insecticides/fungicides on field	Yes	23 (23)
	No	77 (77)
*n* = 97		
Sort spoiled grains before storage	Yes	95 (98)
	No	2 (2)
*n* = 138		
Storage material	In “gotera” ^2^ made from teff straw and mud	5 (3.6)
	In “gotera” made from wood or bamboo	23 (16.6)
	Woven polypropylene bag	83 (60.1)
	Others	27 (19.5)
Insecticides/fungicides in storage	Yes	92 (66.7)
	No	46 (33.3)
Decontamination of spoiled grain	Yes	112 (81.1)
	No	26 (18.9)
*n* = 100		
Duration of storage	1–3 months	14 (14.1)
	4–6 months	50 (49.9)
	7–9 months	12 (11.6)
	10–12 months	24 (24.4)

^1^ mirit zer: is the Amharic version of “improved variety”. ^2^ gotera: stands for a local name for storage made from local materials such as wood and mud.

**Table 4:** Predictors of total FUMs exposure in breast milk samples of the lactating women (*n* = 138).

Predictors	Univariate Analysis		Multivariate Analysis	
	Coefficients	*p*-Values	Coefficients	*p*-Values
Pre-harvest practices				
PC1	−0.31	0.889	0.81	0.736
PC2	0.55	0.818	−1.11	0.699
PC3	−1.87	0.493	−2.06	0.496
Post-harvest practices				
PC1	−0.04	0.984	−0.77	0.744
PC2	5.07	0.078	−6.02	0.068
PC3	0.78	0.745	2.09	0.385
Mycotoxin knowledge				
PC1	−0.12	0.921	0.40	0.762
PC2	0.62	0.672	0.75	0.631
PC3	0.33	0.847	−0.35	0.850
PC4	−1.55	0.427	−2.01	0.382
PC5	2.33	0.283	2.36	0.323
Household maize processing methods				
PC1	1.96	0.227	1.08	0.522
PC2	1.77	0.386	2.43	0.303
FFQ maize	−1.58	0.589	−1.88	0.540
Dietary diversity	−0.01	0.998	0.10	0.961
Age			−1.21	0.034 *
Education level			1.50	0.544
Household food security			0.28	0.907

Censored regression analysis was executed. Total number of observations = 138; left-censored = 117; uncensored = 21. Principal component analysis was used to summarize variables. PC: principal component. FFQ: food frequency questionnaire. Dietary diversity for the women was recorded using the FAO MDD_W. Pre-harvest (PC1: frequency of weeding and use of insecticide in field. PC2: variety common name. PC3: removing residues from previous harvest). Post-harvest (PC1: storage condition. PC2: use of pesticides in storage. PC3: decontamination of molds). Knowledge (PC1: crops prone to mold spoilage and maize prone to mold spoilage. PC2: mycotoxin causes diarrhea. PC3: other health effects of mycotoxin. PC4: wheat prone to mold spoilage. PC5: moisture causes mold in storage). Processing (PC1: make beverages from maize removals. PC2: make other things from maize removals). The asterisks * shows a statistical significant *p*-value < 0.05.

## Data Availability

The data presented in this study are available on request from the corresponding author. The data are not publicly available due to data privacy reasons.
